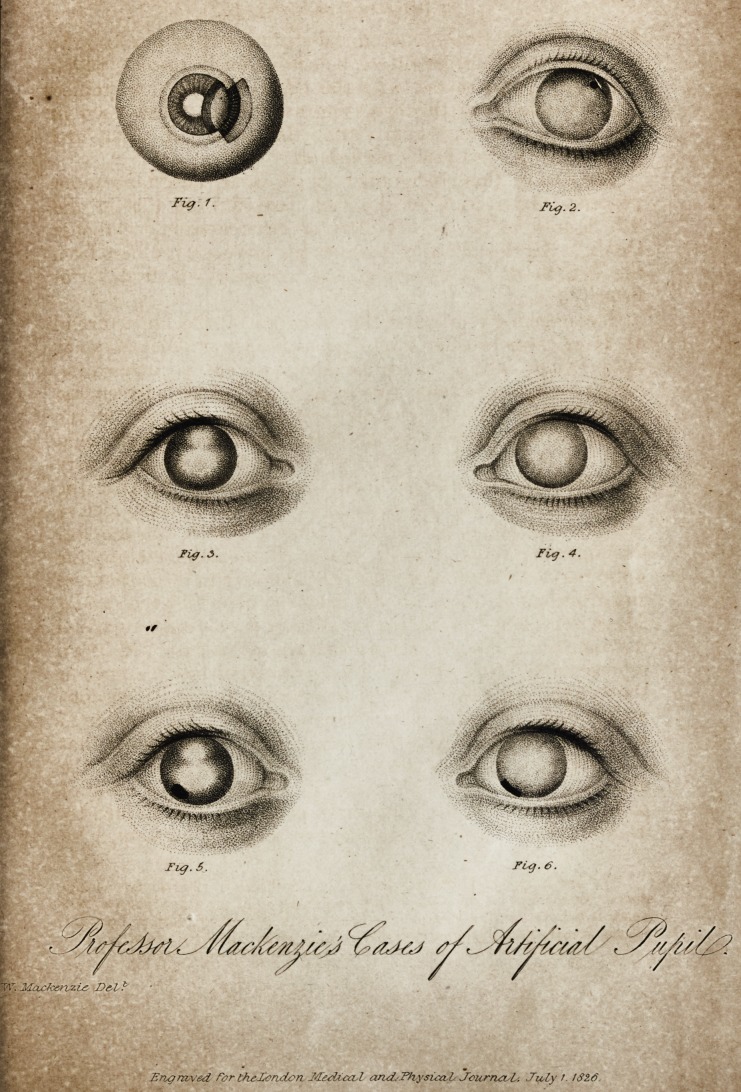# Remarks on Some Representations of the Eye, and on Artificial Pupil

**Published:** 1826-07

**Authors:** William Mackenzie

**Affiliations:** Andersonian Professor of Anatomy and Surgery, and one of the Surgeons to the Glasgow Eye Infirmary.


*Remarks on some Representations of the Eye, and on Artificial Pupil.* By WILLIAM MACKENZIE, Andersonian Professor of Anatomy and Surgery, and one of the Surgeons to the Glasgow Eye Infirmary.*

[WITH AN ENGRAVING.]

ANY representation of anatomical structure to be put into the hands of students, ought to be scrupulously correct, which does not appear to me to be the case with three magnified representations of the eye, engraved from those used in Mr. Mayo's lecture-room, after the plates of Zinn and Soemmerring, and lately published.

The first of these magnified views is an antero-posterior perpendicular section of the eye, derived from the 3d and 4th figures of the eighth plate of Soemmerring's "Icones Oculi." In the following particulars, however, it differs from the figures of Soemmerring; and generally, I think, erroneously.

Soemmerring represents the cartilage of the upper eyelid as three times broader than that of the lower: Mr. Mayo as only twice and a third broader, which is inaccurate. Soemmerring represents the conjunctival fold between the upper eyelid and the eyeball as two-fifths longer, in the perpendicular direction, than that between the lower eyelid and the eyeball: Mr. Mayo represents these folds as very nearly equal, which might lead the student to suppose the upper fold to be scarcely more liable than the lower to receive and conceal foreign substances, blown or driven into the eye. Soemmerring represents the levator oculi as approaching the optic nerve, in its course from the sclerotica to the bottom of the orbit, much more rapidly than the depressor oculi: Mr. Mayo represents these two muscles at very nearly an equal distance from the optic nerve, which is incorrect. Mr. Mayo represents the edge of the cornea as received into a groove of the sclerotica: Soemmerring has not represented the connexion as if formed in this manner, nor does such a mode of connexion exist between these parts. It is well known that the sclerotica overlaps the edge of the cornea; and that the obliquity of the edge of the cornea, arising from this mode of connexion, is such, that the chord of the segment formed by its concave surface is to the chord of the segment formed by

* We are happy to give insertion to Mr. Mackenzie's observations on the anatomy of the Eye: at the same time we think it right to state, that we have reason to believe that the Plate of the Eye used in Mr. Mayo's lecture-room did not aim at more than the general fidelity usual in plans of this description.

38

Series No. 1.

Fig. 1.

Fig. 2.

Fig. 3.

Fig. 4.

Fig. 5.

Fig. 6.

Professor Mackenzie's Cases of Artificial Pupil.

W. Mackenzie Del.t

Engraved for the London Medical and Physical Journal. July 1. 1826.

its convex surface, in the proportion of six to five and a half lines. Mr. Mayo represents the convex surface as more extensive than the concave. Soemmerring represents the antero-posterior diameter of the eye in proportion to the transverse, 29.7 to 27.84 or 106.677 to 100.000: Mr. Mayo as 12.2 to 11.5, or 106.086 to 100.000; and, perhaps, in this point Mr. Mayo is the more correct.

The proportions of the axes of the humours of the eye have been strangely mistaken. If we look into Bartholin's Anatomy, (page 517,) we see a section of the eye which represents the crystalline lens nearly in the middle of the humours; and the error which in Bartholin's figure is exaggerated to the utmost, we find pervading many anatomical books, and of course also optical books, even down to that of Wood, as may be seen by turning to his Optics, (page 123,) where the axis of the aqueous humour is represented as equal to that of the crystalline humour. Oculists have even spoken of pushing the crystalline lens, while yet entire, into the anterior chamber, which is impossible. The lens may be pushed through the pupil, but it will occupy not merely the anterior chamber, but the posterior also, and even part of the space which it occupied when in its natural situation. Soemmerring represents the axis of the crystalline lens to that of the aqueous chambers, as 4.9 to 3.44, or 100.000 to 70.204; Mr. Mayo in the proportion of 1.92 to 1.24, or 100.000 to 64.589. In this point, also, Mr. Mayo is, perhaps, the more correct. Mr. Lawrence, in his notes to Blumenbach's Comparative Anatomy, (page 380,) gives the following as the proportions of the three humours, measured on the axis of the eye, after it had been frozen:—Aqueous, $\frac{5}{22}$; crystalline, $\frac{4}{22}$; vitreous humour, $\frac{15}{22}$. In the recent eye, previously to being frozen, the proportions of the crystalline and aqueous humours are in the reverse proportion to what is here stated, the process of freezing greatly expanding the aqueous humour.

Mr. Mayo represents a triangular space at the circumference of the crystalline lens, and marks it as the canal of Petit; but, in such a section of the eye as this figure is intended to represent, no such space is visible, and is therefore not set down by Soemmerring. Nor is the triangular form assigned by Mr. Mayo to this canal the real form. Soemmerring represents the optic nerve, after its passage through the choroid, as forming a convex projection: Mr. Mayo represents it flat, or even concave.

These discrepancies between Soemmerring's views of this section and Mr. Mayo's, are sufficient to show that the latter has not been very particular in following the great German anatomist.

There are some further remarks regarding this section of the eye, which occur to me, and which I think worthy of attention.

If an antero-posterior perpendicular section of the eye be made exactly through the middle line of that organ, so as to divide it into halves, a nasal and a temporal, they may be very nearly equal, so far as the sclerotica is concerned; but in many respects they will differ remarkably from each other, and it will be impossible, even by representing both the nasal and the temporal surfaces of the section, to make the student sensible of the differences.

1. The nasal half of the eye will contain the entire optic nerve, passing through the sclerotica and choroid; for the optic nerves penetrates through these tunics about the eighth of an inch nearer the nose than the posterior extremity of the common axis of the cornea and globe, and will therefore not be touched at all in such a section.

2. The nasal half of the eye will contain the greater half of the lens, and of course the centre of that body; for the centre of the lens does not correspond to the centre of the cornea, but lies more inwardly, as Varolius appears to have first pointed out.

3. The nasal half of the eye will contain the greater half of the pupil; for the pupil, as Winslow observed, is not in the centre of the iris. The centre of the pupil corresponds, as both Zinn and Haller agree, to the centre of the lens, while that of the iris is in the common axis of the cornea and globe. Dr. Wells, I believe, was the first to show the optical consequence of the centre of the cornea not corresponding to the centres of the pupil and lens; namely, that no ray of light whatsoever can pass unbent from the atmosphere to the retina.

4. The nasal half of the eye will contain the lesser half of the corpus ciliare; for this ring is broader on the temporal side than on the nasal. It follows also that the lesser half of the halo signatus, which is but an impression of the corpus ciliare, and the lesser half of the annulus gangliformis, which covers the corpus ciliare externally, are contained in the nasal half of the bisected eye.

Now, a horizontal section of the eye enables us to represent all the differences which exist between the nasal and temporal hemispheres of the eye, and has therefore been chosen by the younger Soemmerring, as the subject of his splendid Thesis, at Gottingen, in 1818.*

* Detmar Wilhelm Soemmerring, de Oculorum Hominis Animaliumque Sectione Horizontali. Folio.

There is one point in the elder Soemmerring's perpendicular section of the eye, which I should wish to see explained. I have now before me a preparation of the human eye, (and I have added a view of the preparation in Fig. 1,) in which, after cautiously removing the cornea, all except a narrow ring close to its circumference, and removing on one side a small portion also of the sclerotica, I laid hold of the iris with a pair of small forceps, and tore away a portion of the circumference of the iris from its adhesion to the choroid coat. By this means I brought into view the anterior extremities of the ciliary processes, and I now see them forming an indented circle, closely surrounding the edge of the crystalline lens.* But, in the elder Soemmerring's figure, a considerable space is left between the ciliary processes and the lens, and the same space is represented in Mr. Mayo's copy.

No such space is represented in the younger Soemmerring's horizontal section. In Sir Everard Home's section (Phil. Trans. 1822, Plate vi. Fig. 3,) the ciliary processes are even made to overlap considerably the anterior surface of the lens. If the contents of the eye are removed, and the mere tunics examined, I observe, from preparations of the eye now before me, that the ciliary processes appear exactly as the elder Soemmerring and Mr. Mayo have represented them; but, in a section of the tunics and humours, I believe they will appear closely surrounding the edge of the lens.

This is a point of considerable practical importance. I am perfectly assured of the fact, as it exists in the preparation which I have represented in Fig. 1, and Beer has insisted on the same fact in his work on Staphyloma. If this, then, be the constant or natural state of the parts, the needle, in entering the posterior chamber, in the operation of depression or division *per scleroticam,* must be made to pass accurately between two circumferential lines, so as neither to be buried prematurely in the lens, nor to wound the very vascular ciliary processes.

But, if the elder Soemmerring's figure be correct, (and it is with considerable hesitation that I express this doubt, so very high is my respect for that most accurate anatomist,) then there is a space sufficient for the needle to be passed, without much caution; and, what is still more important, an artificial pupil by separation of the iris from the choroid, or by excision

* I always recommend my pupils to make such a preparation of the human eye, and keep it by them, as it shows some of the most interesting points in the surgical anatomy of the organ.

of a portion of the iris close behind the edge of the cornea, might enable a patient to see, even though the lens was opake; an event which has been asserted by Demours to have actually happened, but which Beer has ridiculed as a fable.

In Fig. 2, I have given a sketch of Demours' celebrated case of artificial pupil, a case which has excited more attention and more controversy than perhaps any other case of eye disease upon record.* My own opinion is, that the lens in that case was transparent. The opacity of the greater part of the cornea, and the obliteration of the pupil, which rendered necessary the attempt to form an artificial pupil, took their origin in external inflammation, probably strumous, a disease which rarely has the effect of rendering the lens opake.

That an artificial pupil, although even still closer to the edge of the cornea than that formed by Demours, will not restore vision, if the lens be actually opake, was manifest in the right eye of M. A. (Fig. 3,) in which I formed an artificial pupil by excision of a portion of the iris, as is represented in Figure 5. By this operation an opake lens was brought into view, which impeded vision, and which I divided with the needle three months after; while, in the left eye of the same individual, (Fig. 4,) I formed an artificial pupil by separating a portion of the edge of the iris from the choroid, as is represented in Fig. 6, through which vision was restored without any subsequent operation, the lens in this eye being transparent. Both eyes of this patient had been reduced to the state represented in Figs. 3 and 4, by severe accidental injuries, and consequent inflammation.

Mr. Mayo's second figure is derived from the first figure in Zinn's beautiful fourth Plate; but the copy is objectionable in the following particulars:

1. The sclerotica and choroid are represented of an uniform light brownish colour, which is the true colour of neither tunic.

2. The outline of the eyeball ought to pass over that of the optic nerve, as in Zinn's figure; whereas, in the copy, the outline of the one runs into that of the other.

3. Where two of the ciliary or iridal nerves are represented as dissected through the annulus gangliformis, it might be supposed, from Mr. Mayo's figure, that the whole

* Case of Mr. Sauvages, p. 426 of Demours' Traité des Maladies des Yeux, vol. iii.

thickness of the choroid was removed; whereas, in Zinn's figure, this is seen not to be the case.

4. The other ciliary or iridal nerves are, in Zinn's figure, beautifully represented as sinking into the substance of the annulus gangliformis. In Mr. Mayo's figure, they are represented as if they were cut across.

5. It is well known that there are only four large venæ vorticosæ. Two of them are partially represented in Zinn's figure, with a very small vein between them. But Mr. Mayo has magnified the small intermediate vein to very nearly the same size with the two venæ vorticosæ. These veins are coloured red like the arteries in the other figures. If they had been purple, the student would have at once recognised them to be veins.

Mr. Mayo's third figure is derived from the sixth figure of Soemmerring's fifth Plate; but, in addition, we have an explanatory representation of the structure of the retina, which was not so perfectly understood when Soemmerring published his work.

It is well known that the retina consists of three layers:—1st, the external, lying in contact with the choroid coat, the membrana Jacobiana; 2d, the middle, medullary, or nervous layer; and 3d, the cellulo-vascular, lying in contact with the hyaloid membrane; not, as Mr. Wardrop states it, "the one which is contiguous to the choroid coat being vascular, and the other, which is medullary, being in contact with the capsule of the vitreous humour."* It is also generally known that the membrana Jacobiana is extremely delicate, capable, however, of being raised from the nervous or middle layer, especially when the dissection is performed in water. Mr. Mayo has represented this membrane as thick and stiff, so as to form a strange contrast with the representation given by Dr. Jacob in the twelfth volume of the Medico-Chirurgical Transactions, Plate 9. The nervous layer of the retina may be scraped off with a lancet, and the cellulo-vascular layer left entire; but Mr. Mayo has represented the nervous layer also as raised in the form of a membrane, all which cannot fail to communicate to the student erroneous notions of the structure of the retina.

Mr. Mayo has also represented the limbus luteus and foramen centrale of Soemmerring, as if seen through the membrana Jacobiana; whereas, to see these parts, that layer of the retina must be removed.

Magnified representations of minute parts of the body are

* Morbid Anatomy of the Human Eye, vol. ii. p. 134.

highly useful, and especially magnified representations of so important an organ as the eye. I should not have made these remarks, had I not considered the errors contained in these three figures as too numerous and too important to be passed over, and had I not hoped, by these criticisms, to lead to their correction on a future occasion.

*Spreull's-court, Glasgow; 8th May,* 1826.

## Figures and Tables

**Fig. 1. Fig. 2. Fig. 3. Fig. 4. Fig. 5. Fig. 6. f1:**