# Errata

**Published:** 1826-08

**Authors:** 


					P. 4l?t for " optic nerves," read
??opiic nerve."

				

